# Development of Cytoplasmic Male Sterile IR24 and IR64 Using CW-CMS/*Rf17* System

**DOI:** 10.1186/s12284-016-0097-2

**Published:** 2016-05-11

**Authors:** Kinya Toriyama, Tomohiko Kazama

**Affiliations:** Graduate School of Agricultural Science, Tohoku University, Sendai, 981-8555 Japan

**Keywords:** Cytoplasmic male sterility, Fertility restorer, Hybrid rice

## Abstract

**Background:**

A wild-abortive-type (WA) cytoplasmic male sterility (CMS) has been almost exclusively used for breeding three-line hybrid rice. Many *indica* cultivars are known to carry restorer genes for WA-CMS lines and cannot be used as maintainer lines. Especially elite *indica* cultivars IR24 and IR64 are known to be restorer lines for WA-CMS lines, and are used as male parents for hybrid seed production. If we develop CMS IR24 and CMS IR64, the combination of F_1_ pairs in hybrid rice breeding programs will be greatly broadened.

**Findings:**

For production of CMS lines and restorer lines of IR24 and IR64, we employed Chinese wild rice (CW)-type CMS/*Restorer of fertility 17* (*Rf17*) system, in which fertility is restored by a single nuclear gene, *Rf17*. Successive backcrossing and marker-assisted selection of *Rf17* succeeded to produce completely male sterile CMS lines and fully restored restorer lines of IR24 and IR64. CW-cytoplasm did not affect agronomic characteristics.

**Conclusions:**

Since IR64 is one of the most popular mega-varieties and used for breeding of many modern varieties, the CW-CMS line of IR64 will be useful for hybrid rice breeding.

**Electronic supplementary material:**

The online version of this article (doi:10.1186/s12284-016-0097-2) contains supplementary material, which is available to authorized users.

## Findings

Hybrid rice has an average 15 % to 20 % yield advantage over inbred lines. Hybrid rice planted area accounts for 57.6 % in China, 14.5 % in the USA, 9.4 % in Vietnam, 6.8 % in Bangladesh, 4.3 % in the Philippines, 3.2 % in India, and 0.5 % in Indonesia (Barclay 2010). Most commercial hybrid rice has been developed based on a three-line system, namely A (CMS line), B (maintainer line) and R (restorer line), involving the almost exclusive use of wild-abortive-type CMS (WA-CMS) lines, as it accounted for about 90 % of the rice hybrids produced in China and 100 % of the hybrids developed outside China (Sattari et al. 2007). Although two-line hybrids based on photoperiod- or thermo-sensitive genic male sterile lines are gradually increased in China up to one-third of the total hybrid rice growing area, WA-CMS still predominated (Huang et al. 2014). A major technical handicap in the development of hybrid rice using WA-CMS is a limited source of maintainer lines, as many *indica* elite cultivars are known to carry restorer genes for WA-CMS lines and cannot be used as maintainer lines (Virmani 1994). For example, IR24 and IR64 are restorer lines for WA-CMS lines, and are used as male parents for hybrid seed production (Jing et al. 2001; Cai et al. 2013). Huge efforts had been made to establish maintainer lines for WA-CMS based on *indica* varieties through a maintainer-breeding program at the International Rice Research Institute (Virmani 1994). If we develop CMS-IR24 and CMS-IR64, the combination of F_1_ pairs in hybrid rice breeding programs will be greatly broadened. For production of CMS lines and restorer lines with a nuclear background of elite *indica* cultivars, IR24 or IR64, we employed CW-CMS/*Rf17* system, in which fertility is restored gametophytically by a single nuclear gene, *Rf17* (Fujii and Toriyama 2009).

**Fig. 1:**
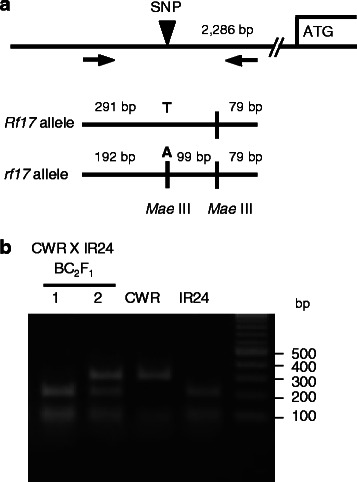
Detection of the *Rf17* allele. **a** An SNP in the promoter region of the *Rf17* gene. **b** PCR-RFLP analysis. PCR products were digested with *Mae* III. The genotype is identified to be *rf17rf17* for a plant in lane 1, and *Rf17rf17* for a plant in lane 2

**Fig. 2:**
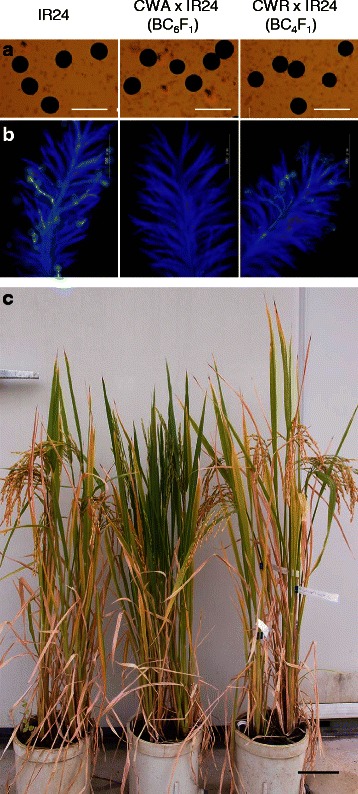
Maintainer line (IR24), CMS line of IR24 (CWA x IR24 BC_6_F_1_) and restorer line of IR24 (CWR x IR24, BC_4_F_1_). **a** Pollen grains stained with I_2_-KI. **b** Pollen tubes on stigma stained with aniline blue. **c** Plants grown in a pot. Bar = 200 μm (**a**), 500 μm (**b**) and 10 cm (**c**)

The Taichung 65 (T65) nuclear background CMS line, CWA with [*cms-CW*]*rf17rf17*, and the restorer line CWR with [*cms-CW*]*Rf17Rf17* (Fujii and Toriyama 2009) were successively backcrossed with IR24 or IR64 by the methods of a biotron breeding system (Ohnishi et al. 2013). The presence or absence of *Rf17* allele was detected by SNP in the promoter region at 2,286-bp upstream of the initiation codon; *Rf17* carried T, while *rf17* carried A (Fujii and Toriyama, 2009). This SNP was found within the recognition sequence (GTNAC) of *Mae* III. PCR-amplification of the promoter region using Primer F: TCGTTCACCACGGTAGATAGACTCAT and Primer R: CCCACATCTTCTCCTTGCATAATCC, followed by digestion with *Mae* III (PCR-RFLP analysis), yielded a 291 bp-band and a 79-bp band for the *Rf17* allele, while yielding bands of 192 bp, 99 bp and 79 bp for the *rf17* allele (Fig. 1). During the backcrossing process, plants with *rf17rf17* were completely sterile, while plants with *Rf17rf17* set seeds (Fig. 2). Pollen grains were observed in the BC_6_F_1_ plants of CWA x IR24 (*rf17rf17*), and in the BC_4_ F_1_ plants of CWR x IR24 (*Rf17rf17*). Pollen grains of CWA x IR24 were morphologically normal and filled with starch but did not germinate on stigma, while pollen grains of CWR x IR24 germinated on stigma in the same manner as IR24 (Fig. 2). This is consistent with the previously observed pollen grains of CWA and CWR, which had the T65 nuclear background (Fujii and Toriyama 2005).

Agronomic characteristics were compared between IR24 and CMS-IR24 (BC_6_F_1_ plants of CWA x IR24) for two years. All experimental materials were transplanted in the paddy field at a spacing of 20 cm between plants within rows and 30 cm between rows. No significant differences were found between the maintainer line and the CMS line in stem length, panicle length, length of exserted panicle, distance between panicle base and auricle of flag leaf, number of panicle per plant, heading date and anthesis time (Table 1, Additional file 1: Table S1). Comparison was also done for IR64 and BC_4_F_1_ or BC_6_F_1_ plants of CWA x IR64. No deleterious characteristics were observed in the CMS-IR64 line, although panicles of the CMS line were slightly larger than those of the maintainer line in the year of 2015 (Table 1, Additional file 1: Table S1).

**Table 1:** Comparison of agronomic characteristics between CMS line and maintainer line in 2014

Year	2014 (*n* = 4)			
Lines	CWA x IR24 (BC_6_F_1_)	IR24	CWA x IR64 (BC_4_F_1_)	IR64
	CMS	Maintainer	CMS	Maintainer
Stem length (cm)	49.9 ± 0.8	50.8 ± 0.8	65.7 ± 1.6	67.4 ± 0.7
Panicle length (cm)	22.6 ± 0.6	22.7 ± 0.3	29.4 ± 0.3	28.9 ± 0.2
Length of exserted panicle (cm)	22.1 ± 0.7	22.6 ± 0.3	29.0 ± 0.1	28.9 ± 0.2
Distance between panicle base and auricle of flag leaf (cm)	−0.5 ± 0.4	0.4 ± 0.3	1.4 ± 1.0	2.9 ± 1.0
Panicle exsertion rate (%)	98	100	100	100
Number of panicle per plant	11.0 ± 0.8	13.8 ± 0.9	19.3 ± 2.3	17.8 ± 0.7
Heading date	Aug. 14	Aug. 14	Aug. 14	Aug. 14
Anthesis time	8:00–11:00	8:00–11:00	8:30–10:30	8:30–10:30

In order to enhance outcrossing, we shook the male parents with a stick to disperse pollen grains daily at peak anthesis for a period of one week at the time of flowering in 2014. The outcrossing rate was 55.5 ± 3.9 % for the CMS-IR24 and 23.9 ± 1.4 % for the CMS-IR64, while the seed setting rate of the maintainer line was 74.3 ± 2.5 % for IR24 and 72.8 ± 2.2 % for IR64 in 2014, and 65.2 ± 1.0 % for IR24 and 79.0 ± 2.7 % for IR64 in 2015.

The seed setting rate of the restorer lines was 82.8 ± 3.9 % for BC_4_F_1_ plants of CWR x IR24 (*Rf17rf17*) when tested in 2014 (Fig. 2). It was 73.5 ± 3.1 % for BC_5_F_2_ plants of CWR x IR24 (*Rf17Rf17*), and 84.4 ± 2.7 % for BC_5_F_2_ plants of CWR x IR64 (*Rf17Rf17*) in 2015. These results demonstrated that the fertility of the restorer line was recovered up to that of the maintainer line.

In conclusion, we produced CMS lines of IR24 or IR64 nuclear background, which did not set any seeds. The fertility is fully recovered by the presence of *Rf17*. CW cytoplasm did not affect agronomic characteristics. The *Rf17* allele was not detected in 69 accessions of the NIAS world rice core collection (Kojima et al. 2005) provided by the National Institute of Agrobiological Sciences Genebank (Tsukuba, Japan) or in any CMS lines so far tested, except for our CWR line (Toriyama et al. 2009). We expect that the CW-CMS/*Rf17* system will be used for production of CMS lines of many *indica* cultivars. Efforts are now in progress to test if the CW-cytoplasm confers CMS in other elite *indica* cultivars. IR64 is one of the most popular mega-varieties and used for breeding of many modern varieties in South-east Asia (Septiningsih et al. 2009). The CW-CMS line of IR64 will be useful for hybrid rice breeding.

## Additional file

Additional file 1: Table S1. Comparison of agronomic characteristics between CMS line and maintainer line in 2015. (XLS 35 kb)

## Abbreviations

CMS: cytoplasmic male sterility
CW-CMS: Chinese wild rice-type CMS
*Rf*: *restorer of fertility*
T65: Taichung 65
WA-CMS: wild-abortive-type CMS
